# Association of estimated glucose disposal rate with risk of future metabolic dysfunction-associated steatotic liver disease and other chronic liver diseases: a prospective cohort study

**DOI:** 10.3389/fmed.2025.1589245

**Published:** 2025-07-02

**Authors:** Yan Li, Xi Ye, Xiaoyu Chen

**Affiliations:** Department of Clinical Pharmacy, Guangxi Academy of Medical Sciences and the People’s Hospital of Guangxi Zhuang Autonomous Region, Nanning, Guangxi, China

**Keywords:** metabolic dysfunction-associated steatotic liver disease, metabolic dysfunction-associated steatohepatitis, insulin resistance, estimated glucose disposal rate, liver cirrhosis, liver neoplasms, metabolic, epidemiology

## Abstract

**Background:**

The incidence of insulin resistance, as determined by estimated glucose disposal rate (eGDR), is associated with various morbidities. The relationship between eGDR and chronic liver diseases remains to be explored. This study examined the association between eGDR and the risk of future metabolic dysfunction-associated steatotic liver disease (MASLD), cirrhosis, liver cancer, and liver-related mortality.

**Method:**

We analyzed data from UK Biobank participants with no history of liver diseases. We calculated the eGDR values for each participant and divided them into four quartile groups based on these values. The primary outcome was MASLD, whereas the secondary outcomes included cirrhosis, liver cancer, and liver-related mortality. We estimated hazard ratios (HRs) and 95% confidence intervals (CIs) using Cox proportional hazard regression models. We used restricted cubic splines models to detect potential non-linear relationships.

**Results:**

This study included data from 290,397 UK Biobank participants who had no history of liver diseases, and the magnetic resonance imaging (MRI)-derived liver proton density fat fraction (PDFF) analysis included 25,810 individuals. Over a median follow-up period of 15.69 years, we identified 3,926 cases of MASLD, 1,553 cases of cirrhosis, 167 cases of liver cancer, and 120 cases of liver-related mortality. After adjusting for multiple variables, higher eGDR levels were significantly associated with a lower risk of MASLD (HR: 0.91, 95% CI: 0.90–0.93), cirrhosis (HR: 0.89, 95% CI: 0.86–0.92), and liver cancer (HR: 0.91, 95% CI: 0.83–1.00). Comparing participants between the lowest and highest quartiles (Q1 and Q4) of eGDR, Q4 had a 47% lower risk of MASLD (HR: 0.53; 95% CI: 0.45–0.63), with similar results for cirrhosis. Moreover, high eGDR levels were associated with a low risk of MASLD based on MRI-derived liver PDFF > 5% (odds ratio: 0.98, 95% CI: 0.97–0.98).

**Conclusion:**

We found a significant inverse correlation between eGDR and MASLD, cirrhosis, and liver cancer. Incorporating eGDR into clinical decision-making can improve the long term follow-up of patients with MASLD.

## Introduction

Chronic liver diseases significantly contribute to global morbidity and mortality, imposing economic burdens and negatively impacting health-related quality of life (1, 2). In June 2023, an international multi-society committee adopted an updated terminology, replacing “fatty liver disease” with steatotic liver disease (3). Within this category, nonalcoholic fatty liver disease has been renamed metabolic dysfunction-associated steatotic liver disease (MASLD) (3). MASLD, affecting approximately 32% of the global population, is a leading cause of cirrhosis and liver cancer, accounting for a substantial proportion of liver-related deaths (3–5). Because no curative treatment currently exists, prioritizing research on modifiable risk factors is essential for disease management (6). Furthermore, progression is associated with substantial health-care costs, socioeconomic losses and reduced quality of life (7).

Most analyses highlight insulin resistance (IR) as a key factor in MASLD pathophysiology, in which impaired glucose metabolism results from diminished target organ responsiveness to insulin (8, 9). This dysfunction promotes systemic lipolysis, resulting in the influx of free fatty acids into the liver and subsequent triglyceride production by hepatocytes (10, 11), and Kupffer cells subsequently phagocytose fat-laden hepatocytes, triggering chronic inflammation and fibrosis (10). Kupffer cells, the resident liver macrophages, are implicated in obesity-induced IR and fatty liver disease (11). Conversely, strategies targeting Kupffer cell function or autophagic processes, including their depletion, can attenuate IR and improve liver health (11). In steatotic livers, reduced Kupffer cell populations were found to be associated with decreased alternative activation and a phenotypic shift toward pro-inflammatory markers (11). This was accompanied by increased autophagy, enhanced lysosomal lipolysis, elevated diacylglycerol levels, activation of protein kinase C epsilon, and marked exacerbation of hepatic insulin resistance (11). Notably, glucose tolerance test results reveal an inverse correlation between IR and fibrosis severity in MASLD patients without diabetes (12), suggesting an independent role for IR in disease progression. The hyperinsulinemic-euglycemic clamp test serves as the gold standard to identify IR; however, this technique is laborious, costly, and therefore impractical in the clinical setting (13). Furthermore, most research have focused on triglyceride-glucose assessment (13) and homeostasis model-based IR evaluation (14), with limited exploration of longitudinal IR indices in relation to chronic liver disease risk. Emerging evidence highlights the estimated glucose disposal rate (eGDR), derived from hemoglobin A1c (HbA1c), hypertension, and waist circumference (WC), as a reliable IR measurement that correlates with hyperinsulinemic-euglycemic clamp assessments (15–17). Importantly, eGDR remains unaffected by renal excretory function, making it a robust clinical parameter (18).

This study aimed to determine the relationship between eGDR and the risk of future MASLD, cirrhosis, liver cancer, and liver-related mortality. To the best of our knowledge, no prior study has systematically examined the relationship between eGDR and the risk of future MASLD. Moreover, research has consistently demonstrated that magnetic resonance imaging (MRI)-derived liver proton density fat fraction (PDFF) is a highly precise and reliable modality for quantifying hepatic fat content, serving as a critical tool in the detection and assessment of liver steatosis (19). Furthermore, whether eGDR is associated with MASLD if defined using MRI-derived PDFF remains unclear. As a further investigation, we also examined the association between eGDR and MRI-derived liver PDFF.

## Methods

### Study design and participants

The UK Biobank enrolled over 500,000 individuals aged 37–73 years between 2006 and 2010 (20). During their baseline visit, participants completed questionnaires, underwent verbal interviews, and physical measurement, and provided biological samples. The first cycle of multimodal imaging studies, including abdominal MRI, was performed in April 2014, involving a subgroup of approximately 100,000 participants (21).

**Figure 1:**
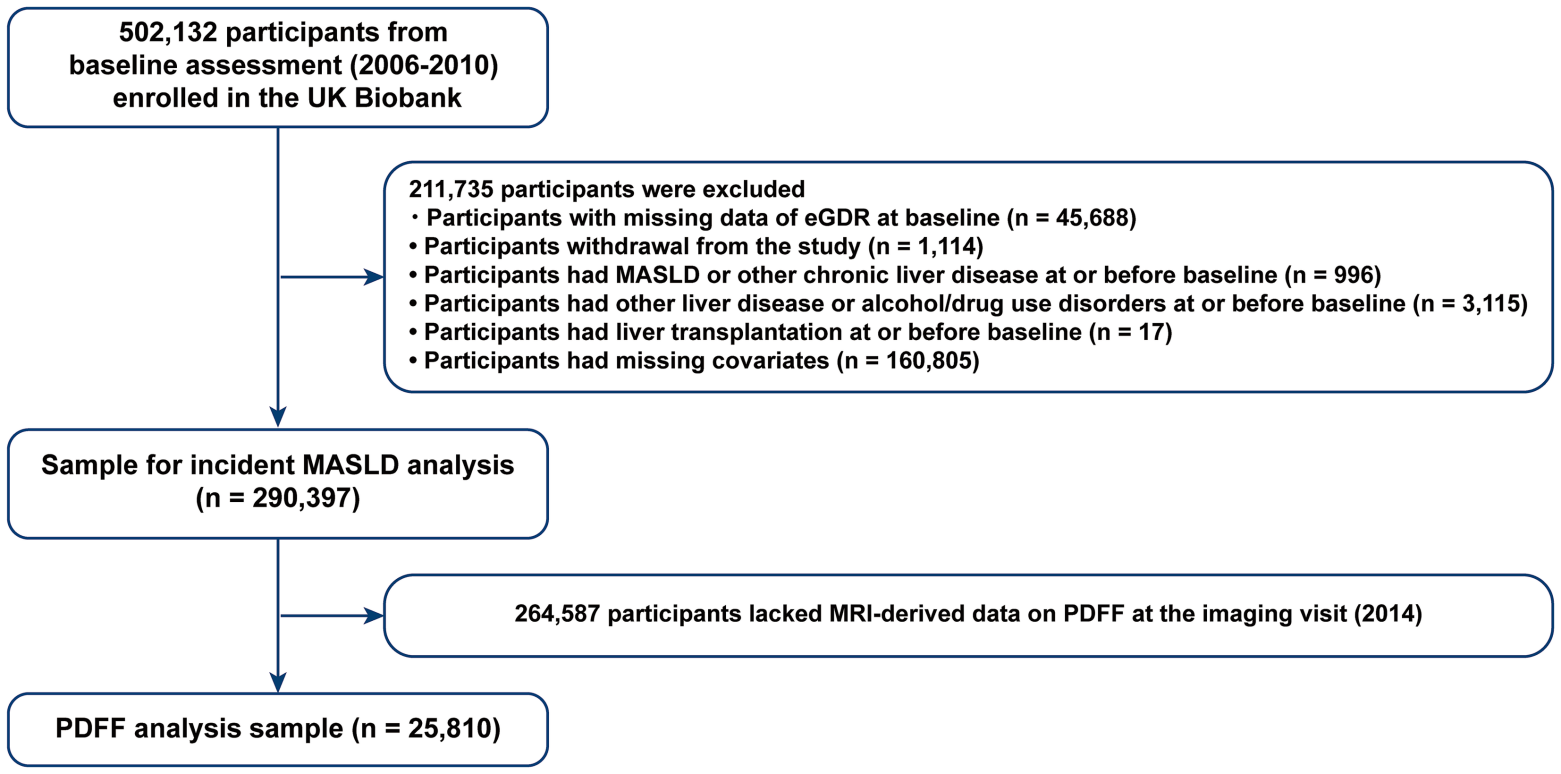
Flowchart of participant selection. eGDR, estimated glucose disposal rate; MASLD, metabolic dysfunction associated steatotic liver disease; PDFF, proton density fat fraction.

From the 502,132 participants, we excluded those with missing eGDR data at baseline (*n* = 45,688), pre-existing MASLD or other chronic liver diseases (*n* = 996), other liver diseases or alcohol/drug use disorders (*n* = 3,115), liver transplantation at or before baseline (*n* = 17) (Supplementary Table S1), and missing covariates (*n* = 160,805) (22, 23). Participants with MASLD (*n* = 531), cirrhosis (*n* = 458), and liver cancer (*n* = 7) were also excluded. Consequently, the analytic sample included 290,397 participants (Figure 1). Details on excluded participants with missing data are provided in Supplementary Table S2. For liver fat content quantification, we further excluded participants without MRI-derived liver PDFF data (*n* = 264,587). The PDFF analysis included 25,810 individuals (Figure 1).

This study was conducted using the UK Biobank data (application number: 283055). This study was approved by the UK National Health Service (NHS) National Research Ethics Service (11/NW/0382) for data collection, analysis, and linkage.

### Exposure assessment

The eGDR was calculated using the following formula: eGDR (mg/kg/min): 21.158 − (0.09 × WC) − (3.407 × hypertension) − (0.551 × HbA1c), in which WC was in cm, hypertension was coded as 1 for yes and 0 for no, and HbA1c was in percentage. The initial assessment of eGDR in the UK Biobank was conducted from 2006 to 2010, with follow-up assessments during repeat visits between 2012 and 2013 (24). Hypertension was defined as self-reported hypertension or use of blood pressure medication when their systolic blood pressure (SBP) was ≥140 mmHg and/or diastolic blood pressure was (DBP) ≥ 90 mmHg.

### Outcome assessment

Data from the NHS, including hospital inpatient and death records, were used to determine study outcomes. The primary outcome was the risk of future MASLD, diagnosed during hospitalization or as a cause of death due to MASLD or metabolic dysfunction-associated steatohepatitis. This outcome was identified through hospital inpatient records, which were coded as either primary or secondary diagnoses (UK Biobank data-field 41,270), and death records, including underlying or contributory causes of death (UK Biobank data-fields 40,001 and 40,002). The outcomes were identified using International Classification of Diseases 10th revision (ICD-10) codes. Specifically, MASLD was identified using ICD-10 codes K76.0 and K75.8 (22). Secondary outcomes included other liver-related diseases, such as cirrhosis, liver cancer, and liver-related mortality (25). Detailed ICD-10 codes for these outcomes are provided in Supplementary Table S3. Follow-up observations began at the date of participant attendance at the assessment center and concluded at the earliest of an outcome event, death, or the last follow-up visit. The duration of follow-up was contingent on the availability of linked health data, with a defined end date of October 31, 2022, August 31, 2022, and May 31, 2022, for participants enrolled in England, Scotland, and Wales, respectively.

Besides liver PDFF, liver MRI was used to measure study outcomes. Detailed descriptions of the MRI detection and analysis protocol are available (21, 26, 27). Body composition analysis was performed using the AMRA Profiler Research tool (27). To determine liver PDFF, nine regions of interest were strategically selected to avoid inhomogeneities, vessels, and bile ducts. For patients with available MRI data, MASLD based on PDFF was defined as an MRI-derived liver PDFF > 5% (28).

### Covariates

Covariates in this study included baseline age, sex (male or female), ethnicity (white or non-white), Townsend deprivation index, and education (college or above, high school or equivalent, or below high school). We also accounted for sleep duration and physical activity (calculated as metabolic equivalent times by summing time spent on each activity weighted by its metabolic equivalent score) (29). Alcohol consumption was categorized as no heavy alcohol consumption (daily alcohol consumption of ≤1 drink for women and ≤2 drinks for men) versus heavy alcohol consumption (29). Smoking status was defined as never (<100 cigarettes in lifetime) or previous/current. Body mass Index (BMI) groups included underweight (BMI < 18.5 kg/m^2^), normal weight (BMI 18.5–25 kg/m^2^), overweight (BMI 25–30 kg/m^2^), and obese (BMI ≥ 30 kg/m^2^). Finally, metabolic syndrome components were also included as covariates: central obesity (WC > 88 cm for women and >102 cm for men), diabetes (self-reported diabetes, or insulin treatment), low HDL (yes: HDL < 1.6 mmol/L, no: HDL ≥ 1.6 mmol/L), and high triglycerides (TG; yes: TG ≥ 2.26 mmol/L, and no: TG < 2.26 mmol/L).

### Statistical analysis

Baseline characteristics were presented as means (standard deviations [SDs]) or medians (interquartile ranges [IQRs]) for continuous variables, and as counts (percentages) for categorical variables. We compared continuous variables between groups using one-way analysis of variance or Kruskal–Wallis tests, whereas categorical variables were compared using the Chi-square test.

We used Cox proportional hazard models to estimate the hazard ratios (HRs) and 95% confidence intervals (CIs) associated with eGDR and primary or secondary outcomes. Participants were categorized into four groups based on their eGDR quartile scores. The crude incidence rate, determined by comparing the lowest eGDR quartile with the others, was calculated to determine the association between eGDR and the risk of future of primary and secondary outcomes.

Schoenfeld residual tests were used to verify that each variable in the model complies with the proportional hazards assumption. Time-to-event calculations was performed from the date of enrollment at the assessment center and ended at the first occurrence of the study outcome, death, loss to follow-up, or censorship. Model 1 was an unadjusted model. Model 2 was adjusted for age, sex, ethnicity, education, Townsend deprivation index, physical activity, smoking status, alcohol consumption, and sleep duration. Model 3 was further adjusted for BMI, central obesity, diabetes, low HDL, and high TG. In the fully adjusted model, restricted cubic splines (RCS) were used to evaluate the dose–response relationships between eGDR and the risk of future MASLD, cirrhosis, liver cancer, and liver-related deaths. Additionally, the association between eGDR and PDFF was estimated using multivariable logistic regression models, with outcomes presented as odds ratios (ORs) and their 95% CIs.

We performed a stratified analysis by age (<65 years vs. ≥65 years), sex, central obesity (yes vs. no), physical activity (enough activity vs. not meeting enough activity), smoking status (never, previous/current), alcohol consumption (heavy alcohol consumption vs. no heavy alcohol consumption), diabetes (yes vs. no), low HDL (yes vs. no), and high TG (yes vs. no). Furthermore, sensitivity analyses were performed. Individuals with heavy alcohol consumption at baseline were excluded. Multiple imputations were applied across five datasets to handle missing covariate data. Landmark analysis was extended to 5 years to reduce reverse causation bias. Furthermore, the missing values in covariates were addressed using multivariate imputation by chained equations. Only covariates with missing data were imputed. We maintained five imputations, generating five imputed datasets to account for uncertainty. Five imputations are often cited as sufficient for moderate levels of missingness. This approach preserves the integrity of the dataset while minimizing potential bias owing to missing values. All statistical analyses were performed using R version 4.2.1 (R Foundation for Statistical Computing, Vienna, Austria).

## Results

### Baseline characteristics

During a median follow-up of 15.69 (14.94–16.40) years, 3,926 (1.35%) incidents of MASLD, 1,553 (0.54%) incidents of cirrhosis, 167 (0.06%) incidents of liver cancer, and 120 (0.04%) deaths due to liver-related diseases were recorded. Baseline eGDR was 8.45 ± 2.45, and quartiles of eGDR were categorized into Q1 (5.26 ± 0.99), Q2 (7.33 ± 0.63), Q3 (9.77 ± 0.56), and Q4 (11.44 ± 0.54). The detailed description can be found in Table 1. Characteristics of participants with PDFF data are shown in Supplementary Table S4, and the characteristics of participants in the multiple imputation analysis are presented in Supplementary Table S5. Mean age, SBP, DBP, WC, and HbA1c significantly decreased with increasing levels of eGDR (all *p* < 0.001). Additionally, participants with higher eGDR levels tend to have low HDL and high TG.

**Table 1:** Baseline characteristics of participants stratified by quartiles of estimated glucose disposal rate.

Characteristic	Overall	Quartiles of eGDR				
		Quartile 1	Quartile 2	Quartile 3	Quartile 4	*p* value
No.	290,397	72,621	72,591	72,588	72,597	
eGDR, mean (SD)	8.45 (2.45)	5.26 (0.99)	7.33 (0.63)	9.77 (0.56)	11.44 (0.54)	<0.001
Follow-up, years, median (IQR)	15.69 (14.94, 16.40)	15.57 (14.72, 16.32)	15.67 (14.94, 16.36)	15.71 (14.98, 16.42)	15.77 (15.06, 16.49)	<0.001
Baseline age, years, mean (SD)	56.20 (8.11)	58.76 (7.41)	58.15 (7.58)	54.80 (8.10)	53.10 (7.96)	<0.001
Sex, male, *n* (%)	133,916 (46%)	55,433 (76%)	31,702 (44%)	36,814 (51%)	9,967 (14%)	<0.001
Ethnicity, *n* (%)						<0.001
Non-white	13,691 (4.7%)	3,445 (4.7%)	3,186 (4.4%)	3,907 (5.4%)	3,153 (4.3%)	
White	276,706 (95%)	69,176 (95%)	69,405 (96%)	68,681 (95%)	69,444 (96%)	
Education, *n* (%)						<0.001
College or above	137,147 (47%)	31,681 (44%)	32,814 (45%)	35,319 (49%)	37,333 (51%)	
High school or equivalent	112,943 (39%)	26,850 (37%)	28,092 (39%)	28,722 (40%)	29,279 (40%)	
Less than high school	40,307 (14%)	14,090 (19%)	11,685 (16%)	8,547 (12%)	5,985 (8.2%)	
Townsend deprivation index, mean (SD)	−1.45 (2.99)	−1.30 (3.08)	−1.59 (2.92)	−1.38 (3.03)	−1.55 (2.93)	
Physical activity, *n* (%)						<0.001
Enough activity	177,868 (61%)	40,303 (55%)	46,562 (64%)	43,675 (60%)	47,328 (65%)	
Not meeting enough activity	112,529 (39%)	32,318 (45%)	26,029 (36%)	28,913 (40%)	25,269 (35%)	
Smoking status, *n (%)*						<0.001
Never	131,987 (45%)	39,705 (55%)	31,748 (44%)	32,925 (45%)	27,609 (38%)	
Previous/current	158,410 (55%)	32,916 (45%)	40,843 (56%)	39,663 (55%)	44,988 (62%)	
Alcohol consumption, *n* (%)						<0.001
Heavy alcohol consumption	155,731 (54%)	39,277 (54%)	39,861 (55%)	37,017 (51%)	39,576 (55%)	
No heavy alcohol consumption	134,666 (46%)	33,344 (46%)	32,730 (45%)	35,571 (49%)	33,021 (45%)	
Sleep duration, mean (SD)	7.17 (1.07)	7.17 (1.16)	7.18 (1.08)	7.13 (1.06)	7.19 (0.99)	
BMI, mean (SD), kg/m^2^						<0.001
Underweight (<18.5)	1,462 (0.5%)	0 (0%)	125 (0.2%)	209 (0.3%)	1,128 (1.6%)	
Normal weight (18.5 to <25)	97,125 (33%)	2,387 (3.3%)	27,525 (38%)	15,688 (22%)	51,525 (71%)	
Overweight (25 to <30)	124,180 (43%)	31,824 (44%)	32,661 (45%)	40,642 (56%)	19,053 (26%)	
Obese ≥ 30	67,630 (23%)	38,410 (53%)	12,280 (17%)	16,049 (22%)	891 (1.2%)	
WC, mean (SD), cm	89.96 (13.42)	104.09 (9.56)	87.85 (11.24)	91.97 (7.98)	75.93 (5.89)	<0.001
SBP, mean (SD), mmHg	137.38 (18.56)	150.17 (15.61)	149.24 (16.01)	127.26 (10.62)	121.69 (10.45)	<0.001
DBP, mean (SD), mmHg	82.17 (10.14)	88.78 (9.60)	86.68 (9.03)	78.14 (6.93)	74.54 (7.12)	<0.001
HbA1c, mean (SD), %	5.43 (0.60)	5.72 (0.88)	5.40 (0.51)	5.38 (0.40)	5.23 (0.32)	<0.001
Metabolic syndrome, *n* (%)						
Central obesity	93,754 (32%)	47,376 (65%)	17,671 (24%)	28,566 (39%)	141 (0.2%)	<0.001
Diabetes	14,079 (4.8%)	9,205 (13%)	2,657 (3.7%)	1,809 (2.5%)	408 (0.6%)	<0.001
Low HDL	198,566 (68%)	63,219 (87%)	46,061 (63%)	55,124 (76%)	34,162 (47%)	<0.001
High TG	63,283 (22%)	26,802 (37%)	14,726 (20%)	17,157 (24%)	4,598 (6.3%)	<0.001

BMI, body mass index; DBP, diastolic blood pressure; eGDR, estimated glucose disposal rate; HbA1c, glycosylated hemoglobin A1c; HDL, high-density lipoprotein; SBP, systolic blood pressure; SD, standard deviation; TG, triglyceride; WC, waist circumference.

### Association of baseline eGDR and risk of future MASLD, cirrhosis, liver cancer, and liver-related mortality

The relationship between eGDR and risk of future MASLD, cirrhosis, liver cancer, and liver-related deaths based on a Cox regression analysis is shown in Table 2. Considering sociodemographic variables (age, sex, ethnicity, Townsend deprivation index, and educational level), lifestyle factors (BMI, sleep, physical activity, alcohol consumption, and smoking status), and medical conditions (central obesity, diabetes, low HDL, and high TG), an increase of 1.0 SD in eGDR is associated with a 9% decreased risk for MASLD (HR 0.91, 95% CI 0.90–0.93), 11% decreased risk for cirrhosis (HR 0.89, 95% CI 0.86–0.92), and 9% decreased risk for liver cancer (HR 0.91, 95% CI 0.83–1.00). However, eGDR did not appear to be associated with liver-related mortality. When continuous eGDR data were divided into quartiles, the risk of MASLD associated with Q4 was 47% lower than Q1 (HR 0.53, 95% CI 0.45–0.63), with a significant trend observed (*p* < 0.001). Compared with Q1, Q4 was associated with a 49% reduction in the risk of cirrhosis.

**Table 2:** Association of estimated glucose disposal rate for risk of future MASLD, cirrhosis, liver cancer, and liver-related mortality.

	Total *N*	No. of events (Incident rate, %)	Model 1		Model 2		Model 3	
			HR (95% CI)	*p* value	HR (95% CI)	*p* value	HR (95% CI)	*p* value
MASLD								
Continues								
Per SD increase	290,397	3,926 (1.352)	0.78 (0.77, 0.79)	<0.001	0.77 (0.76, 0.78)	<0.001	0.91 (0.90, 0.93)	<0.001
Quartiles								
Q1	72,621	1,854 (2.553)	Ref		Ref		Ref	
Q2	72,591	912 (1.256)	0.49 (0.45, 0.53)	<0.001	0.47 (0.43, 0.51)	<0.001	0.91 (0.83, 0.99)	0.03
Q3	72,588	872 (1.201)	0.47 (0.43, 0.51)	<0.001	0.43 (0.39, 0.47)	<0.001	0.73 (0.67, 0.80)	<0.001
Q4	72,597	288 (0.397)	0.15 (0.14, 0.17)	<0.001	0.13 (0.11, 0.15)	<0.001	0.53 (0.45, 0.63)	<0.001
*P* for trend				<0.001		<0.001		<0.001
Cirrhosis								
Continues								
Per SD increase	290,397	1,553 (0.535)	0.75 (0.74, 0.77)	<0.001	0.80 (0.78, 0.82)	<0.001	0.89 (0.86, 0.92)	<0.001
Quartiles								
Q1	72,621	809 (1.114)	Ref		Ref		Ref	
Q2	72,591	336 (0.463)	0.41 (0.36, 0.47)	<0.001	0.52 (0.45, 0.59)	<0.001	0.77 (0.66, 0.89)	<0.001
Q3	72,588	272 (0.375)	0.34 (0.29, 0.38)	<0.001	0.43 (0.37, 0.50)	<0.001	0.64 (0.55, 0.75)	<0.001
Q4	72,597	136 (0.187)	0.17 (0.14, 0.20)	<0.001	0.27 (0.22, 0.33)	<0.001	0.51 (0.40, 0.65)	<0.001
*P* for trend				<0.001		<0.001		<0.001
Liver cancer								
Continues								
Per SD increase	290,397	167 (0.058)	0.69 (0.66, 0.73)	<0.001	0.77 (0.72, 0.83)	<0.001	0.91 (0.83, 1.00)	0.049
Quartiles								
Q1	72,621	112 (0.154)	Ref		Ref		Ref	
Q2	72,591	25 (0.034)	0.22 (0.14, 0.34)	<0.001	0.38 (0.24, 0.59)	<0.001	0.65 (0.40, 1.06)	0.084
Q3	72,588	17 (0.023)	0.15 (0.09, 0.25)	<0.001	0.30 (0.18, 0.50)	<0.001	0.54 (0.31, 0.94)	0.031
Q4	72,597	13 (0.018)	0.12 (0.07, 0.21)	<0.001	0.47 (0.25, 0.90)	0.022	1.16 (0.52, 2.56)	0.721
*P* for trend				<0.001		<0.001		0.188
Liver-related mortality								
Continues								
Per SD increase	290,397	120 (0.041)	0.68 (0.65, 0.71)		0.76 (0.71, 0.82)		0.90 (0.81, 1.01)	0.085
Quartiles								
Q1	72,621	83 (0.114)	Ref		Ref		Ref	
Q2	72,591	20 (0.028)	0.23 (0.14, 0.38)	<0.001	0.42 (0.26, 0.70)	<0.001	0.80 (0.47, 1.39)	0.436
Q3	72,588	10 (0.014)	0.12 (0.06, 0.22)	<0.001	0.24 (0.12, 0.48)	<0.001	0.48 (0.24, 0.98)	0.043
Q4	72,597	7 (0.01)	0.08 (0.04, 0.17)	<0.001	0.38 (0.16, 0.90)	0.028	1.28 (0.46, 3.55)	0.639
*P* for trend				<0.001		<0.001		0.194

Levels of significance: *p* < 0.05 (Cox regression model). Model 1, unadjusted. Model 2, adjusted for age, sex, ethnicity, education, Townsend deprivation index, physical activity, smoking status, alcohol consumption, and sleep duration. Model 3, model 2 + further adjusted for BMI, central obesity, diabetes, low HDL, and high TG. BMI, body mass index; CI, confidence interval; HDL, high density lipoprotein; HR, hazard ratio; MASLD, metabolic dysfunction-associated steatotic liver disease; SD, standard deviation; TG, triglyceride.

**Figure 2:**
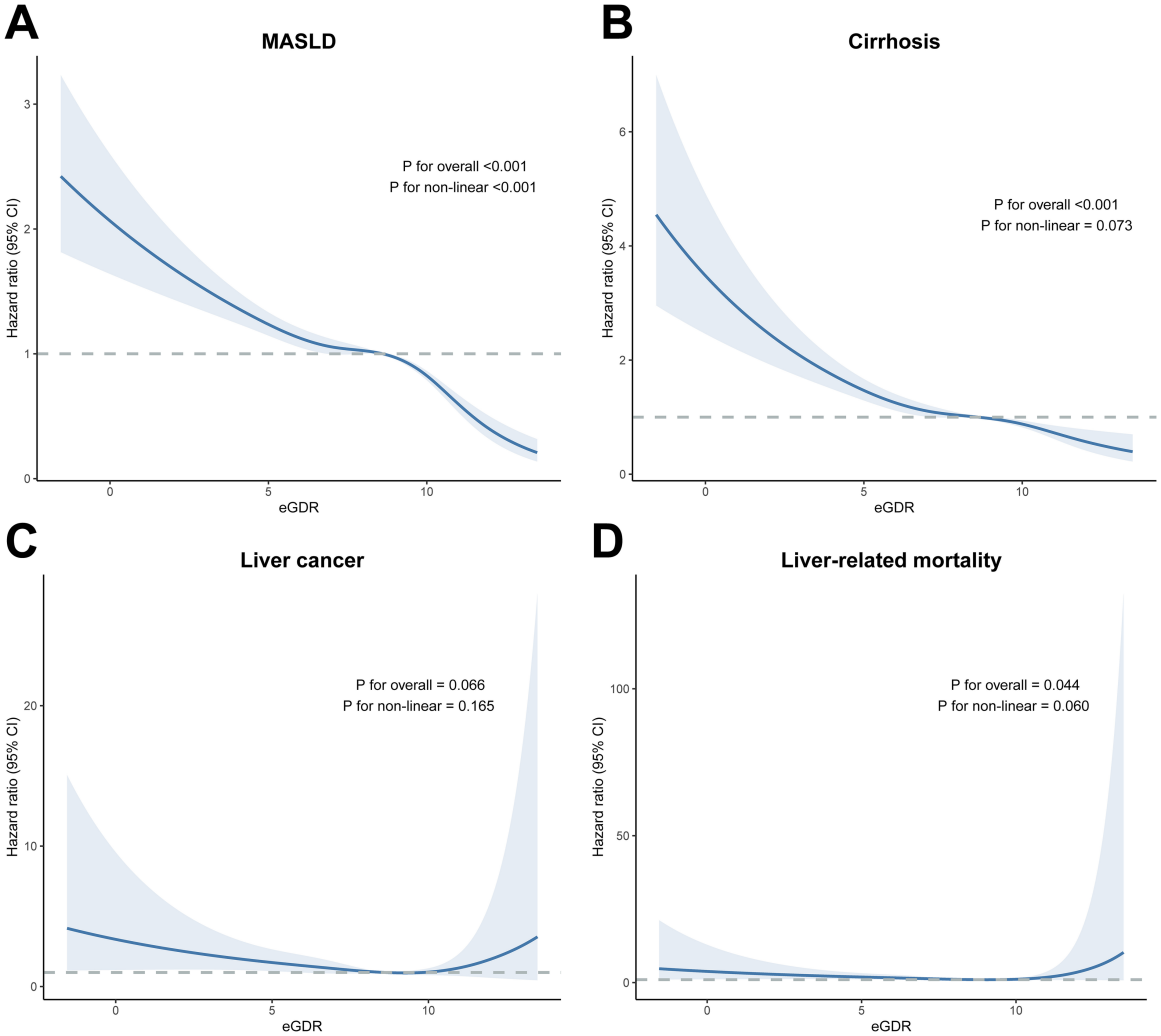
Dose–response association of eGDR with the risk of future MASLD, cirrhosis, liver cancer, and **(A)** MASLD, **(B)** cirrhosis, **(C)** liver cancer, **(D)** liver-related mortality. The blue lines indicate the hazard ratios, and the shaded areas indicate the 95% confidence intervals. Three knots were used to fit the data using a restricted cubic spline. Models were adjusted for age, sex, ethnicity, education, Townsend deprivation index, physical activity, smoking status, alcohol consumption, sleep duration, BMI, central obesity, diabetes, low HDL, and high TG. Levels of significance: *p* < 0.05. eGDR, estimated glucose disposal rate; BMI, body mass index; HDL, high density lipoprotein; MASLD, metabolic dysfunction-associated steatotic liver disease; TG, triglyceride.

A non-linear relationship was observed between continuous eGDR and MASLD risk (*P* for non-linear < 0.001), with MASLD risk declining monotonically across a wide range of eGDR. The influence of eGDR also decreased monotonically as eGDR increased (Figure 2). Further analysis using RCS suggested an almost linear relationship between continuous eGDR and the risks of cirrhosis (*P* for non-linear = 0.073), liver cancer (*P* for non-linear = 0.165), and liver-related mortality (*P* for non-linear = 0.060).

### Association of baseline eGDR and proton density fat fraction

The relationship between eGDR and MASLD risk (defined as PDFF >5%) is presented in Table 3. In the fully adjusted logistic regression model, a 1 SD increase in eGDR significantly lowered the risk of MASLD (OR 0.98, 95% CI 0.97–0.98). Additionally, higher eGDR levels (Q2–Q4) were associated with progressively lower odds of incident MASLD compared to Q1 (OR 0.93, 95% CI 0.92–0.94 for Q2; OR 0.91, 95% CI 0.89–0.92 for Q3; OR 0.86, 95% CI 0.84–0.88 for Q4).

**Table 3:** Association between estimated glucose disposal rate and MASLD defined as PDFF > 5%.

	Total *N*	No. of events (Incident rate, %)	Model 1		Model 2		Model 3	
			OR (95% CI)	*p* value	OR (95% CI)	*p* value	OR (95% CI)	*p* value
Continues								
Per SD increase	25,810	231 (0.895)	0.94 (0.94, 0.94)	<0.001	0.94 (0.94, 0.94)	<0.001	0.98 (0.97, 0.98)	<0.001
Quartiles								
Q1	6,453	95 (1.472)	Ref		Ref		Ref	
Q2	6,454	77 (1.193)	0.83 (0.82, 0.84)	<0.001	0.83 (0.82, 0.84)	<0.001	0.93 (0.92, 0.94)	<0.001
Q3	6,450	45 (0.698)	0.80 (0.79, 0.81)	<0.001	0.80 (0.78, 0.81)	<0.001	0.91 (0.89, 0.92)	<0.001
Q4	6,453	14 (0.217)	0.66 (0.65, 0.67)	<0.001	0.66 (0.65, 0.67)	<0.001	0.86 (0.84, 0.88)	<0.001
*P* for trend				<0.001		<0.001		<0.001

Levels of significance: *p* < 0.05 (logistic regression model). Model 1, unadjusted. Model 2, adjusted for age, sex, ethnicity, education, Townsend deprivation index, physical activity, smoking status, alcohol consumption, and sleep duration. Model 3, model 2 + further adjusted for BMI, central obesity, diabetes, low HDL, and high TG. BMI, body mass index; CI, confidence interval; HDL, high density lipoprotein; HR, hazard ratio; MASLD, metabolic dysfunction-associated steatotic liver disease; SD, standard deviation; TG, triglyceride.

### Additional analysis

**Figure 3:**
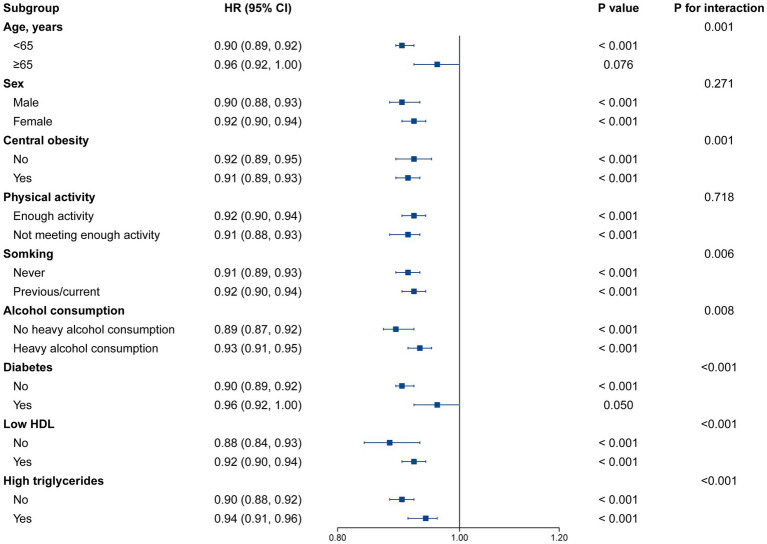
Stratified analysis for the association between eGDR and MASLD risk. Models were adjusted for age, sex, ethnicity, education, Townsend deprivation index, physical activity, smoking status, alcohol consumption, sleep duration, BMI, central obesity, diabetes, low HDL, and high TG. Levels of significance: *p* < 0.05. eGDR, estimated glucose disposal rate; BMI, body mass index; HDL, high density lipoprotein; MASLD, metabolic dysfunction-associated steatotic liver disease; TG, triglyceride.

A stronger relationship between eGDR and risk of future MASLD was observed in participants aged <65 years, those with central obesity, those who never smoked, those without heavy alcohol consumption, those without diabetes, and those with normal HDL or TG levels with a higher eGDR score (P for interaction < 0.05; Figure 3). A stronger association between eGDR and risk of future cirrhosis was observed in those aged <65 years, women, those with sufficient physical activity, those without heavy alcohol consumption, and those with normal HDL levels (P for interaction <0.05; Supplementary Figure S1). The results of stratified analyses for liver cancer and liver-related mortality are presented in Supplementary Figures S2, S3, with no significant interaction observed for most subgroups. We conducted multiple sensitivity analyses (Supplementary Tables S6–S8) and found that the results remained consistent. When we evaluated the cohort excluding individuals with heavy alcohol consumption, used multiple imputations of covariates, and used landmark 5-year analysis, the association between eGDR and the risk of MASLD, cirrhosis, and liver cancer was modestly intensified and remained significant. Furthermore, the association between eGDR and liver-related mortality were also significant under these sensitivity analyses.

## Discussion

Our comprehensive analysis of UK Biobank data revealed that eGDR is significantly associated with an increased risk of future MASLD. Specifically, a lower eGDR is significantly correlated with a higher risk of future MASLD, cirrhosis, and liver cancer. Thus, implementing preventive and intervention strategies targeting eGDR may reduce the risk of MASLD. We found that eGDR was more strongly associated with MASLD risk in participants aged < 65 years, those with central obesity, those who never smoked, those without heavy alcohol consumption, those without diabetes, and those with normal HDL or TG levels. However, the effect of eGDR on liver cancer and liver-related mortality does not differ significantly across various subgroups.

MASLD affects a substantial proportion of the general population, significantly elevating the risk of various diseases and adversely impacting health outcomes. Identifying potential prognostic and risk factors is crucial for better management of this population. Patients with MASLD often exhibit IR, disrupted glucose and lipid metabolism, and increased inflammation (30). The use of eGDR in the general population has been widely recognized as a straightforward and reliable method to detect IR and assess cardiovascular risk (15, 31). Notably, significant progress has been made in using these surrogate biomarkers for MASLD screening. Previous studies have demonstrated the relationship between eGDR and MASLD. In a study including 150 participants with type 1 diabetes and 30 participants with MASLD, eGDR and metabolic syndrome were significantly associated with the presence of the disease (32). Furthermore, another study involving 151 adults with type 1 diabetes discovered that MASLD is more likely to occur in individuals with lower eGDR (33). Compared to these previous studies, our study revealed a longitudinal association between eGDR and the risk of future MASLD in a cohort that includes participants without type 1 diabetes. These results further indicated that eGDR is associated with other chronic liver diseases, such as cirrhosis and liver cancer.

IR is influenced by various factors, including inflammation, oxidative stress, abnormal insulin metabolism signaling pathways, and mitochondrial dysfunction (34). IR significantly contributes to MASLD progression; thus, assessing IR indicators is crucial for predicting MASLD incidence. By suppressing gluconeogenesis in the liver and enhancing *de novo* lipogenesis, insulin promotes fat oxidation (35, 36). However, in IR, although the suppression of gluconeogenesis is defective, hepatic lipogenesis remains unrestrained. Although IR is associated with increased hepatic steatosis, its exact mechanism remains unclear. A main point of contention is whether insulin still directly regulates hepatic lipogenesis in this state (30). Evidence suggests that blocking the insulin/IRS/AKT pathway reduces liver lipid deposition in IR, indicating that insulin remains crucial in regulating lipid metabolism (30). Additionally, hepatic glucose metabolism bypassing the effects of insulin may be important in lipogenesis, as could peripheral IR in other tissues affecting liver lipid composition (35). Furthermore, a previous study revealed that low eGDR is associated with liver fibrosis, independent of glycemic control and glucose-lowering treatment (37). This highlights the significant role of IR in MASLD development and its progression to more severe steatosis and fibrosis, likely by increasing hepatic free fatty acid influx, enhancing *de novo* lipogenesis, and inhibiting fatty acid oxidation (37). Given that traditional methods for assessing IR status are invasive and costly, calculating eGDR based solely on WC, HbA1c, and hypertension offers a more practical and feasible option for large-scale clinical applications. Notably, individuals without diabetes are significantly more affected by WC and hypertension than by HbA1c. This can be attributed to the relatively low HbA1c levels in participants without diabetes. Nevertheless, WC, hypertension, and HbA1c each contribute significantly to eGDR.

Our subgroup analysis highlights the complex interplay between eGDR and the risk of future MASLD, demonstrating how various demographic and metabolic factors shape disease susceptibility. MASLD is prevalent among adults and increasingly recognized in younger populations, emphasizing the need for age-specific reference values for fasting glucose-insulin metabolism to enhance risk stratification, especially in children with obesity (38–40). IR and obesity are key drivers of MASLD, as IR promotes hepatic lipid accumulation and metabolic dysregulation, whereas obesity exacerbates IR through adipose tissue dysfunction and chronic inflammation, accelerating hepatic steatosis and the risk of advanced liver disease (38, 41). The relationship between MASLD and metabolic complications is further influenced by visceral adipose tissue accumulation and intramyocellular triglyceride deposition, both of which contribute to metabolic dysfunction (38). Although low HDL cholesterol is frequently linked to MASLD and IR, normal HDL levels do not necessarily exclude underlying IR (42). Importantly, MASLD can develop independently of diabetes, with IR remaining a central factor in disease pathogenesis (42–44). Additionally, smoking and alcohol consumption contribute to MASLD progression, and as they exert direct hepatotoxic effects, IR further compounds liver fat accumulation and inflammation, amplifying disease severity (45, 46). Addressing these interconnected metabolic and lifestyle factors is critical for effective MASLD prevention and management. Although IR is strongly linked to MASLD and its progression, it is not necessarily associated with liver-related mortality in all cases. IR is a major factor in the development of MASLD, which can progress to steatohepatitis, cirrhosis, and hepatocellular carcinoma, all of which can contribute to liver-related mortality (47–49). However, the relationship between IR and liver-related mortality is complex, further influenced by factors, such as genetic predisposition, environmental factors, and disease severity (47–49).

This study had several limitations. First, as observational research, it cannot establish causality. However, our findings remain robust despite the positive associations observed in landmark sensitivity analyses conducted over 5 years. Second, despite adjusting for numerous covariates, residual confounding cannot be eliminated, a common limitation in observational studies. Third, relying on hospital inpatient data and death registration records for MASLD identification poses a risk of bias, particularly concerning the increased risk of hospitalization and MASLD diagnosis among individuals with severe comorbidities. Nevertheless, MRI-derived liver PDFF helped identify undiagnosed cases of MASLD, although the results were not significantly different. Fourth, although HbA1c levels are typically measured using standard methods, certain conditions, such as iron deficiency anemia, erythropoietin administration, and splenectomy, may influence measurement accuracy. Fifth, despite their generally accuracy for identifying MASLD, ICD-10 codes have inherent limitations, particularly in distinguishing MASLD from other liver diseases or conditions with similar symptoms (50, 51). These limitations may lead to misclassification and an underestimation of the actual prevalence in specific populations. Therefore, a more comprehensive approach to diagnosis and management is necessary to ensure accurate disease identification and better patient outcomes. However, our study examined the relationship between eGDR and MASLD defined using MRI-derived liver PDFF and found consistently robust results. Finally, the ethnic and racial homogeneity of UK Biobank participants limit the generalizability of our findings, necessitating further validation in more diverse cohorts.

## Conclusion

Overall, eGDR was significantly correlated with MASLD, cirrhosis, and liver cancer. Individuals with lower eGDR levels are at a higher risk for MASLD, cirrhosis, and liver cancer. The results revealed that eGDR could facilitate clinical decision-making for patients with MASLD during long-term follow-up by assisting clinicians in identifying early signs of the disease.

## Data Availability

The original contributions presented in the study are included in the article/Supplementary material, further inquiries can be directed to the corresponding author.
