# Supplementary material for: Association of estimated glucose disposal rate with risk of future metabolic dysfunction-associated steatotic liver disease and other chronic liver diseases: a prospective cohort study
Source: Front Med (Lausanne). 2025 Jul 2;12:1589245. doi: 10.3389/fmed.2025.1589245 (PMC12263629; doi:10.3389/fmed.2025.1589245)

Supplementary material

Supplementary Tables

Supplemental Table S1. ICD-10 codes for diseases used as exclusion criteria.

Supplemental Table S2. Detailed information on missing covariates.

Supplemental Table S3. ICD-10 codes utilized in the UKB for defining outcomes.

Supplemental Table S4. Baseline characteristics of participants in PDFF analysis.

Supplemental Table S5. Baseline characteristics of participants in multiple imputations.

Supplemental Table S6. Association of estimated glucose disposal rate for incident MASLD, cirrhosis, liver cancer, and liver-related mortality after excluding individuals with heavy alcohol consumption.

Supplemental Table S7. Association of estimated glucose disposal rate for incident MASLD, cirrhosis, liver cancer, and liver-related mortality using multiple imputation.

Supplemental Table S8. Association of estimated glucose disposal rate for incident MASLD, cirrhosis, liver cancer, and liver-related mortality using landmark 5-year analysis.

Supplementary Figures

Supplemental Figure S1. Stratified analysis for the association between eGDR and cirrhosis risk.

Supplemental Figure S2. Stratified analysis for the association between eGDR and liver cancer risk.

Supplemental Figure S3. Stratified analysis for the association between eGDR and liver-related mortality risk.

Supplemental Table S1. ICD-10 codes for diseases used as exclusion criteria.

Disease at or before baseline	ICD-10 code
**Other liver diseases**	
Alcoholic liver disease (ALD)	K70
Viral hepatitis	B16, B17, B18, B19
Autoimmune liver disease (autoimmune hepatitis, primary biliary cholangitis, primary sclerosing cholangitis)	K83.0, K74.3, K75.4
Hemochromatosis	E83.1
Wilson	E83.0
Alpha-1-antitrypsin deficiency	E88.0
Budd-Chiari	I82.0, K76.5
Chronic hepatitis, unspecified	K73.9, K73.2
Secondary or unspecified biliary cirrhosis	K74.4, K74.5
**Alcohol/drug use disorder**	
Codes associated with alcohol use disorder	F10
Codes associated with somatic consequences of alcohol (except ALD)	E24.4, G62.1, I42.6, K29.2, G31.2, G72.1, K85.2, K86.0, T51.0, T51.9, Y57.3, X65, Z50.2, Z71.4, Z72.1
Codes associated with drug use disorders except nicotine/caffeine	F11, F12, F13, F14, F16, F18, F19
**Liver transplantation**	Z94.4

Supplemental Table S2. Detailed information on missing covariates.

Variables	N*	Missing rate (%)
Ethnicity	0	0
Education	5294	1.05%
Townsend deprivation index	546	0.11%
Physical activity	99030	19.72%
Smoking status	860	0.17%
Alcohol consumption	10785	2.15%
Sleep duration	628	0.13%
BMI	587	0.12%
Central obesity	0	0
Diabetes	368	0.07%
Low HDL	42509	8.47%
High triglycerides	198	0.04%

*N represents the number of missing responses.

Abbreviation: BMI, body mass index; HDL, high-density lipoprotein.

Supplemental Table S3. ICD-10 codes utilized in the UKB for defining outcomes.

Outcomes	ICD-10 code	Description
MASLD	K76.0	Fatty (change of) liver, not elsewhere classified
	K75.8	Other specified inflammatory liver diseases
Cirrhosis	K70.2	Alcoholic fibrosis and sclerosis of liver
	K70.3	Alcoholic cirrhosis of liver
	K70.4	Alcoholic hepatic failure
	K74.0	Hepatic fibrosis
	K74.1	Hepatic sclerosis
	K74.2	Hepatic fibrosis with hepatic sclerosis
	K74.6	Other and unspecified cirrhosis of liver
	K76.6	Portal hypertension
	I85.0	Oesophageal varices with bleeding
	I85.9	Oesophageal varices without bleeding
Liver cancer	C22.0	Liver cell carcinoma
Liver-related mortality	K70	Alcoholic liver disease
	K71	Toxic liver disease
	K72	Hepatic failure, not elsewhere classified
	K73	Chronic hepatitis, not elsewhere classified
	K74	Fibrosis and cirrhosis of liver
	K75	Other inflammatory liver diseases
	K76	Other diseases of liver
	K77	Liver disorders in diseases classified elsewhere
	I85	Oesophageal varices
	C22.0	Liver cell carcinoma

Supplemental Table S4. Baseline characteristics of participants in PDFF analysis.

Characteristic	Overall	Quartiles of eGDR				
		Quartile 1	Quartile 2	Quartile 3	Quartile 4	P value
No.	25,810	6,453	6,454	6,450	6,453	
eGDR, mean (SD)	8.92 (2.34)	5.74 (0.87)	8.06 (0.83)	10.27 (0.40)	11.63 (0.46)	
Follow-up, years, median (IQR)	15.80 (15.08, 16.48)	15.72 (15.01, 16.42)	15.75 (15.04, 16.42)	15.82 (15.11, 16.49)	15.98 (15.17, 16.55)	<0.001
Baseline age, years, mean (SD)	54.86 (7.52)	57.48 (7.03)	56.04 (7.24)	53.87 (7.45)	52.06 (7.19)	<0.001
Sex, male, n (%)	12,219 (47%)	5,104 (79%)	3,102 (48%)	3,320 (51%)	693 (11%)	<0.001
Ethnicity, n (%)						0.445
Non-white	762 (3.0%)	180 (2.8%)	192 (3.0%)	208 (3.2%)	182 (2.8%)	
White	25,048 (97%)	6,273 (97%)	6,262 (97%)	6,242 (97%)	6,271 (97%)	
Education, n (%)						<0.001
College or above	15,050 (58%)	3,650 (57%)	3,677 (57%)	3,835 (59%)	3,888 (60%)	
High school or equivalent	9,353 (36%)	2,289 (35%)	2,395 (37%)	2,306 (36%)	2,363 (37%)	
Less than high school	1,407 (5.5%)	514 (8.0%)	382 (5.9%)	309 (4.8%)	202 (3.1%)	
Townsend deprivation index, mean (SD)	-1.92 (2.70)	-1.95 (2.67)	-1.97 (2.66)	-1.84 (2.75)	-1.90 (2.72)	0.104
Physical activity, n (%)						<0.001
Enough activity	15,880 (62%)	3,659 (57%)	3,975 (62%)	4,009 (62%)	4,237 (66%)	
Not meeting enough activity	9,930 (38%)	2,794 (43%)	2,479 (38%)	2,441 (38%)	2,216 (34%)	
Smoking status, n (%)						<0.001
Never	10,398 (40%)	3,084 (48%)	2,676 (41%)	2,506 (39%)	2,132 (33%)	
Previous/current	15,412 (60%)	3,369 (52%)	3,778 (59%)	3,944 (61%)	4,321 (67%)	
Alcohol consumption, n (%)						<0.001
Heavy alcohol consumption	15,159 (59%)	3,965 (61%)	3,713 (58%)	3,675 (57%)	3,806 (59%)	
No heavy alcohol consumption	10,651 (41%)	2,488 (39%)	2,741 (42%)	2,775 (43%)	2,647 (41%)	
Sleep duration, mean (SD)	7.17 (0.95)	7.17 (1.00)	7.17 (0.95)	7.14 (0.95)	7.21 (0.91)	
BMI, mean (SD), kg/m^2						<0.001
Underweight (<18.5)	116 (0.4%)	0 (0%)	14 (0.2%)	2 (<0.1%)	100 (1.5%)	
Normal weight (18.5 to <25)	10,177 (39%)	450 (7.0%)	2,461 (38%)	2,143 (33%)	5,123 (79%)	
Overweight (25 to <30)	10,986 (43%)	3,521 (55%)	2,618 (41%)	3,647 (57%)	1,200 (19%)	
Obese ≥30	4,531 (18%)	2,482 (38%)	1,361 (21%)	658 (10%)	30 (0.5%)	
WC, mean (SD), cm	87.80 (12.51)	99.95 (8.88)	88.72 (12.69)	88.42 (4.82)	74.09 (5.12)	<0.001
SBP, mean (SD), mmHg	134.88 (17.68)	149.58 (14.38)	142.88 (16.46)	125.14 (9.10)	120.75 (10.58)	<0.001
DBP, mean (SD), mmHg	81.42 (9.90)	89.13 (8.79)	84.71 (8.84)	77.10 (6.60)	74.16 (7.13)	<0.001
HbA1c, mean (SD), %	5.35 (0.46)	5.53 (0.63)	5.36 (0.44)	5.31 (0.34)	5.19 (0.31)	<0.001
Metabolic syndrome, n (%)						

Central obesity	6,322 (24%)	3,188 (49%)	1,776 (28%)	1,357 (21%)	1 (<0.1%)	<0.001
Diabetes	667 (2.6%)	393 (6.1%)	183 (2.8%)	69 (1.1%)	22 (0.3%)	<0.001
Low HDL	16,994 (66%)	5,459 (85%)	4,239 (66%)	4,560 (71%)	2,736 (42%)	<0.001
High TG	4,854 (19%)	2,129 (33%)	1,323 (20%)	1,137 (18%)	265 (4.1%)	<0.001

BMI, body mass index; DBP, diastolic blood pressure; eGDR, estimated glucose disposal rate; HbA1c, glycosylated hemoglobin A1c; HDL, high-density lipoprotein; SBP, systolic blood pressure; SD, standard deviation; TG, triglyceride WC waist circumference.

Supplemental Table S5. Baseline characteristics of participants in multiple imputations.

Characteristic	Overall	Quartiles of eGDR				
		Quartile 1	Quartile 2	Quartile 3	Quartile 4	P value
No.	451,202	112,806	112,795	112,808	112,793	
eGDR, mean (SD)	8.39 (2.46)	5.21 (1.00)	7.26 (0.60)	9.68 (0.59)	11.41 (0.55)	<0.001
Follow-up, years, median (IQR)	15.69 (14.95, 16.40)	15.57 (14.72, 16.31)	15.67 (14.94, 16.36)	15.71 (15.00, 16.42)	15.77 (15.08, 16.49)	<0.001
Baseline age, years, mean (SD)	56.57 (8.09)	59.03 (7.35)	58.53 (7.50)	55.26 (8.11)	53.47 (8.02)	<0.001
Sex, male, n (%)	201,192 (45%)	83,151 (74%)	47,645 (42%)	54,148 (48%)	16,248 (14%)	<0.001
Ethnicity, n (%)						<0.001
Non-white	24,694 (5.5%)	6,457 (5.7%)	5,762 (5.1%)	7,040 (6.2%)	5,435 (4.8%)	
White	426,508 (95%)	106,349 (94%)	107,033 (95%)	105,768 (94%)	107,358 (95%)	
Education, n (%)						<0.001
College or above	198,485 (44%)	45,179 (40%)	47,221 (42%)	50,993 (45%)	55,092 (49%)	
High school or equivalent	170,739 (38%)	39,659 (35%)	42,312 (38%)	43,693 (39%)	45,075 (40%)	
Less than high school	81,978 (18%)	27,968 (25%)	23,262 (21%)	18,122 (16%)	12,626 (11%)	
Townsend deprivation index, mean (SD)	-1.34 (3.06)	-1.12 (3.16)	-1.50 (2.99)	-1.26 (3.10)	-1.48 (2.99)	<0.001
Physical activity, n (%)						<0.001
Enough activity	274,791 (61%)	61,702 (55%)	71,677 (64%)	67,414 (60%)	73,998 (66%)	
Not meeting enough activity	176,411 (39%)	51,104 (45%)	41,118 (36%)	45,394 (40%)	38,795 (34%)	
Smoking status, n (%)						<0.001
Never	202,575 (45%)	60,692 (54%)	48,577 (43%)	50,667 (45%)	42,639 (38%)	
Previous/current	248,627 (55%)	52,114 (46%)	64,218 (57%)	62,141 (55%)	70,154 (62%)	
Alcohol consumption, n (%)						<0.001
Heavy alcohol consumption	288,575 (64%)	69,308 (61%)	73,375 (65%)	69,633 (62%)	76,259 (68%)	
No heavy alcohol consumption	162,627 (36%)	43,498 (39%)	39,420 (35%)	43,175 (38%)	36,534 (32%)	
Sleep duration, mean (SD)	7.16 (1.10)	7.16 (1.20)	7.17 (1.10)	7.12 (1.09)	7.17 (1.02)	<0.001
BMI, mean (SD), kg/m2						<0.001
Underweight (<18.5)	2,341 (0.5%)	1 (<0.1%)	158 (0.1%)	399 (0.4%)	1,783 (1.6%)	
Normal weight (18.5 to <25)	147,641 (33%)	3,568 (3.2%)	41,400 (37%)	24,276 (22%)	78,397 (70%)	
Overweight (25 to <30)	191,511 (42%)	47,451 (42%)	52,499 (47%)	60,535 (54%)	31,026 (28%)	
Obese ≥30	109,709 (24%)	61,786 (55%)	18,738 (17%)	27,598 (24%)	1,587 (1.4%)	
WC, mean (SD), cm	90.15 (13.49)	104.39 (9.66)	87.89 (10.84)	92.06 (8.74)	76.27 (6.03)	<0.001
SBP, mean (SD), mmHg	137.84 (18.66)	150.34 (15.72)	149.92 (15.91)	127.99 (11.41)	121.96 (10.40)	<0.001
DBP, mean (SD), mmHg	82.22 (10.14)	88.62 (9.67)	86.77 (9.04)	78.35 (7.09)	74.61 (7.10)	<0.001
HbA1c, mean (SD), %	5.46 (0.62)	5.76 (0.91)	5.42 (0.52)	5.40 (0.42)	5.24 (0.32)	<0.001
Metabolic syndrome, n (%)						

Central obesity	152,031 (34%)	76,157 (68%)	27,353 (24%)	48,187 (43%)	334 (0.3%)	<0.001
Diabetes	24,277 (5.4%)	15,607 (14%)	4,438 (3.9%)	3,495 (3.1%)	737 (0.7%)	<0.001
Low HDL	310,497 (69%)	97,638 (87%)	72,621 (64%)	84,938 (75%)	55,300 (49%)	<0.001
High TG	99,688 (22%)	41,568 (37%)	23,094 (20%)	26,905 (24%)	8,121 (7.2%)	<0.001

BMI, body mass index; DBP, diastolic blood pressure; eGDR, estimated glucose disposal rate; HbA1c, glycosylated hemoglobin A1c; HDL, high-density lipoprotein; SBP, systolic blood pressure; SD, standard deviation; TG, triglyceride WC waist circumference.

Supplemental Table S6. Association of estimated glucose disposal rate for incident MASLD, cirrhosis, liver cancer, and liver-related mortality after excluding individuals with heavy alcohol consumption.

	Total N	No. of events (Incident rate)	Model 1		Model 2		Model 3	
			HR (95% CI)	P value	HR (95% CI)	P value	HR (95% CI)	P value
**MASLD**								
Continues								
Per SD increase	155731	1775 (1.14)	0.77 (0.75, 0.78)	<0.001	0.77 (0.75, 0.78)	<0.001	0.89 (0.87, 0.92)	<0.001
Quartiles								
Q1	39277	867 (2.207)	Ref		Ref		Ref	
Q2	39861	426 (1.069)	0.48 (0.43, 0.54)	<0.001	0.48 (0.43, 0.55)	<0.001	0.95 (0.83, 1.09)	0.492
Q3	37017	342 (0.924)	0.42 (0.37, 0.47)	<0.001	0.41 (0.36, 0.47)	<0.001	0.67 (0.58, 0.77)	<0.001
Q4	39576	140 (0.354)	0.16 (0.13, 0.19)	<0.001	0.15 (0.12, 0.18)	<0.001	0.56 (0.44, 0.71)	<0.001
P for trend				<0.001		<0.001		<0.001
**Cirrhosis**								
Continues								
Per SD increase	155731	886 (0.569)	0.74 (0.72, 0.76)	<0.001	0.79 (0.77, 0.81)	<0.001	0.85 (0.82, 0.89)	<0.001
Quartiles								
Q1	39277	478 (1.217)	Ref		Ref		Ref	
Q2	39861	195 (0.489)	0.40 (0.34, 0.47)	<0.001	0.53 (0.45, 0.63)	<0.001	0.72 (0.59, 0.89)	0.002
Q3	37017	136 (0.367)	0.30 (0.25, 0.36)	<0.001	0.39 (0.32, 0.48)	<0.001	0.55 (0.45, 0.69)	<0.001
Q4	39576	77 (0.195)	0.16 (0.13, 0.20)	<0.001	0.29 (0.22, 0.37)	<0.001	0.43 (0.31, 0.61)	<0.001
P for trend				<0.001		<0.001		<0.001
**Liver cancer**								
Continues								
Per SD increase	155731	101 (0.065)	0.69 (0.65, 0.72)	<0.001	0.76 (0.70, 0.82)	<0.001	0.86 (0.75, 0.98)	0.022
Quartiles								
Q1	39277	73 (0.186)	Ref		Ref		Ref	
Q2	39861	14 (0.035)	0.19 (0.11, 0.33)	<0.001	0.34 (0.19, 0.61)	<0.001	0.60 (0.32, 1.13)	0.115
Q3	37017	7 (0.019)	0.10 (0.05, 0.22)	<0.001	0.21 (0.10, 0.46)	<0.001	0.36 (0.16, 0.82)	0.015
Q4	39576	7 (0.018)	0.10 (0.04, 0.21)	<0.001	0.50 (0.21, 1.22)	0.13	1.26 (0.43, 3.68)	0.677
P for trend				<0.001		<0.001		0.093
**Liver-related mortality**								
Continues								
Per SD increase	155731	70 (0.045)	0.68 (0.64, 0.71)	<0.001	0.73 (0.68, 0.79)	<0.001	0.80 (0.69, 0.92)	0.002
Quartiles								

Q1	39277	54 (0.137)	Ref		Ref		Ref	
Q2	39861	10 (0.025)	0.18 (0.09, 0.35)	<0.001	0.32 (0.16, 0.63)	0.001	0.58 (0.27, 1.24)	0.162
Q3	37017	3 (0.008)	0.06 (0.02, 0.18)	<0.001	0.12 (0.04, 0.38)	<0.001	0.21 (0.06, 0.70)	0.011
Q4	39576	3 (0.008)	0.05 (0.02, 0.17)	<0.001	0.27 (0.07, 0.96)	0.043	0.76 (0.17, 3.40)	0.725
P for trend				<0.001		<0.001		0.02

Levels of significance: $p < 0.05$ (Cox regression model).

CI, confidence interval; HR, hazard ratio; MASLD, metabolic dysfunction-associated steatotic liver disease; SD, standard deviation.

Model 1: unadjusted.

Model 2: adjusted for age, sex, ethnicity, education, townsend deprivation index, physical activity, smoking status, alcohol consumption, and sleep duration.

Model 3: model 2 + further adjusted for BMI, central obesity, diabetes, low HDL, and high TG.

Supplemental Table S7. Association of estimated glucose disposal rate for incident MASLD, cirrhosis, liver cancer, and liver-related mortality using multiple imputation.

	Total N	No. of events (Incident rate)	Model 1		Model 2		Model 3	
			HR (95% CI)	P value	HR (95% CI)	P value	HR (95% CI)	P value
**MASLD**								
Continues								
Per SD increase	451202	6593 (1.461)	0.78 (0.78, 0.79)	<0.001	0.77 (0.76, 0.78)	<0.001	0.91 (0.90, 0.92)	<0.001
Quartiles								
Q1	112806	3089 (2.738)	Ref		Ref		Ref	
Q2	112795	1466 (1.3)	0.47 (0.44, 0.50)	<0.001	0.45 (0.42, 0.48)	<0.001	0.86 (0.80, 0.93)	<0.001
Q3	112808	1539 (1.364)	0.49 (0.46, 0.53)	<0.001	0.45 (0.42, 0.48)	<0.001	0.74 (0.69, 0.79)	<0.001
Q4	112793	499 (0.442)	0.16 (0.15, 0.18)	<0.001	0.13 (0.12, 0.15)	<0.001	0.51 (0.45, 0.58)	<0.001
P for trend				<0.001		<0.001		<0.001
**Cirrhosis**								
Continues								
Per SD increase	451202	2630 (0.583)	0.77 (0.75, 0.78)	<0.001	0.81 (0.80, 0.82)	<0.001	0.90 (0.88, 0.92)	<0.001
Quartiles								
Q1	112806	1336 (1.184)	Ref		Ref		Ref	
Q2	112795	576 (0.511)	0.43 (0.39, 0.47)	<0.001	0.54 (0.49, 0.60)	<0.001	0.80 (0.71, 0.90)	<0.001
Q3	112808	455 (0.403)	0.34 (0.31, 0.38)	<0.001	0.43 (0.39, 0.48)	<0.001	0.63 (0.56, 0.71)	<0.001
Q4	112793	263 (0.233)	0.20 (0.17, 0.22)	<0.001	0.31 (0.27, 0.36)	<0.001	0.57 (0.48, 0.69)	<0.001
P for trend				<0.001		<0.001		<0.001
**Liver cancer**								
Continues								
Per SD increase	451202	267 (0.059)	0.69 (0.66, 0.71)	<0.001	0.77 (0.73, 0.81)	<0.001	0.88 (0.82, 0.95)	0.001
Quartiles								
Q1	112806	180 (0.16)	Ref		Ref		Ref	
Q2	112795	39 (0.035)	0.22 (0.15, 0.31)	<0.001	0.36 (0.26, 0.52)	<0.001	0.54 (0.36, 0.81)	0.003
Q3	112808	28 (0.025)	0.16 (0.10, 0.23)	<0.001	0.30 (0.20, 0.45)	<0.001	0.47 (0.30, 0.73)	<0.001
Q4	112793	20 (0.018)	0.11 (0.07, 0.18)	<0.001	0.43 (0.26, 0.72)	0.001	0.75 (0.40, 1.40)	0.36
P for trend				<0.001		<0.001		0.006
**Liver-related mortality**								
Continues								
Per SD increase	451202	190 (0.042)	0.67 (0.65, 0.70)	<0.001	0.75 (0.71, 0.80)	<0.001	0.90 (0.83, 0.99)	0.023
Quartiles								

Q1	112806	125 (0.111)	Ref		Ref		Ref	
Q2	112795	35 (0.031)	0.27 (0.19, 0.40)	<0.001	0.47 (0.32, 0.68)	<0.001	0.87 (0.56, 1.34)	0.531
Q3	112808	18 (0.016)	0.14 (0.08, 0.23)	<0.001	0.27 (0.17, 0.45)	<0.001	0.53 (0.31, 0.91)	0.021
Q4	112793	12 (0.011)	0.09 (0.05, 0.16)	<0.001	0.37 (0.19, 0.71)	0.002	1.01 (0.46, 2.22)	0.973
P for trend				<0.001		<0.001		0.109

Levels of significance: p <0.05 (Cox regression model).

Model 1: unadjusted.

Model 2: adjusted for age, sex, ethnicity, education, townsend deprivation index, physical activity, smoking status, alcohol consumption, and sleep duration.

Model 3: model 2 + further adjusted for BMI, central obesity, diabetes, low HDL, and high TG.

BMI, body mass index; CI, confidence interval; HDL, high density lipoprotein; HR, hazard ratio; MASLD, metabolic dysfunction-associated steatotic liver disease; SD, standard deviation; TG, triglyceride.

Supplemental Table S8. Association of estimated glucose disposal rate for incident MASLD, cirrhosis, liver cancer, and liver-related mortality using landmark 5-year analysis.

	Total N	No. of events (Incident rate)	Model 1		Model 2		Model 3	
			HR (95% CI)	P value	HR (95% CI)	P value	HR (95% CI)	P value
**MASLD**								
Continues								
Per SD increase	289856	3385 (1.168)	0.79 (0.78, 0.80)	<0.001	0.78 (0.76, 0.79)	<0.001	0.92 (0.90, 0.94)	<0.001
Quartiles								
Q1	72323	1556 (2.151)	Ref		Ref		Ref	
Q2	72464	785 (1.083)	0.49 (0.45, 0.53)	<0.001	0.48 (0.44, 0.52)	<0.001	0.91 (0.83, 1.01)	0.068
Q3	72490	774 (1.068)	0.47 (0.43, 0.51)	<0.001	0.45 (0.41, 0.49)	<0.001	0.75 (0.68, 0.83)	<0.001
Q4	72579	270 (0.372)	0.15 (0.14, 0.17)	<0.001	0.14 (0.13, 0.17)	<0.001	0.57 (0.48, 0.68)	<0.001
P for trend				<0.001		<0.001		<0.001
**Cirrhosis**								
Continues								
Per SD increase	290081	1237 (0.426)	0.75 (0.73, 0.76)	<0.001	0.78 (0.76, 0.80)	<0.001	0.87 (0.84, 0.90)	<0.001
Quartiles								
Q1	72471	659 (0.909)	Ref		Ref		Ref	
Q2	72522	267 (0.368)	0.41 (0.36, 0.47)	<0.001	0.48 (0.42, 0.56)	<0.001	0.72 (0.61, 0.86)	<0.001
Q3	72525	209 (0.288)	0.34 (0.29, 0.38)	<0.001	0.39 (0.33, 0.45)	<0.001	0.58 (0.48, 0.69)	<0.001
Q4	72563	102 (0.141)	0.17 (0.14, 0.20)	<0.001	0.22 (0.17, 0.28)	<0.001	0.42 (0.32, 0.56)	<0.001
P for trend				<0.001		<0.001		<0.001
**Liver cancer**								
Continues								
Per SD increase	290361	131 (0.045)	0.68 (0.65, 0.72)	<0.001	0.75 (0.70, 0.81)	<0.001	0.89 (0.80, 0.99)	0.0319
Quartiles								
Q1	72602	93 (0.128)	Ref		Ref		Ref	
Q2	72582	16 (0.022)	0.22 (0.14, 0.34)	<0.001	0.29 (0.17, 0.50)	<0.001	0.51 (0.28, 0.93)	0.027
Q3	72583	12 (0.017)	0.15 (0.09, 0.25)	<0.001	0.26 (0.14, 0.47)	<0.001	0.48 (0.25, 0.94)	0.032
Q4	72594	10 (0.014)	0.12 (0.07, 0.21)	<0.001	0.44 (0.21, 0.93)	0.031	1.09 (0.44, 2.74)	0.852
P for trend				<0.001		<0.001		0.151
**Liver-related mortality**								
Continues								
Per SD increase	290397	120 (0.041)	0.67 (0.64, 0.71)	<0.001	0.75 (0.70, 0.80)	<0.001	0.87 (0.77, 0.99)	0.033
Quartiles								

Q1	72621	83 (0.114)	Ref		Ref		Ref	
Q2	72591	20 (0.028)	0.23 (0.14, 0.38)	<0.001	0.36 (0.20, 0.64)	<0.001	0.68 (0.37, 1.26)	0.22
Q3	72588	10 (0.014)	0.12 (0.06, 0.22)	<0.001	0.22 (0.11, 0.47)	<0.001	0.44 (0.20, 0.97)	0.041
Q4	72597	7 (0.01)	0.08 (0.04, 0.17)	<0.001	0.39 (0.16, 0.98)	0.046	1.26 (0.41, 3.84)	0.684
P for trend				<0.001		<0.001		0.158

Levels of significance: $p < 0.05$ (Cox regression model).

Model 1: unadjusted.

Model 2: adjusted for age, sex, ethnicity, education, townsend deprivation index, physical activity, smoking status, alcohol consumption, and sleep duration.

Model 3: model 2 + further adjusted for BMI, central obesity, diabetes, low HDL, and high TG.

BMI, body mass index; CI, confidence interval; HDL, high density lipoprotein; HR, hazard ratio; MASLD, metabolic dysfunction-associated steatotic liver disease; SD, standard deviation; TG, triglyceride.

Supplemental Figure S1. Stratified analysis for the association between eGDR and cirrhosis risk. Models were adjusted for age, sex, ethnicity, education, townsend deprivation index, physical activity, smoking status, alcohol consumption, sleep duration, BMI, central obesity, diabetes, low HDL, and high TG. Levels of significance: $p < 0.05$. eGDR, estimated glucose disposal rate; BMI, body mass index; HDL, high density lipoprotein; MASLD, metabolic dysfunction-associated steatotic liver disease; TG, triglyceride.

Subgroup	HR (95% CI)	P value	P for interaction
**Age, years**			0.033
<65	0.87 (0.84, 0.90)	< 0.001	
≥65	0.96 (0.90, 1.03)	0.242	
**Sex**			0.040
Male	0.89 (0.86, 0.93)	< 0.001	
Female	0.88 (0.84, 0.93)	< 0.001	
**Central obesity**			0.490
No	0.94 (0.90, 0.98)	0.009	
Yes	0.85 (0.82, 0.89)	< 0.001	
**Physical activity**			0.033
Enough activity	0.87 (0.84, 0.91)	< 0.001	
Not meeting enough activity	0.90 (0.87, 0.94)	< 0.001	
**Somking**			0.169
Never	0.89 (0.85, 0.92)	< 0.001	
Previous/current	0.90 (0.86, 0.94)	< 0.001	
**Alcohol consumption**			0.003
No heavy alcohol consumption	0.85 (0.82, 0.89)	< 0.001	
Heavy alcohol consumption	0.93 (0.89, 0.97)	< 0.001	
**Diabetes**			0.188
No	0.89 (0.86, 0.92)	< 0.001	
Yes	0.88 (0.83, 0.95)	< 0.001	
**Low HDL**			0.002
No	0.87 (0.82, 0.93)	< 0.001	
Yes	0.89 (0.86, 0.92)	< 0.001	
**High triglycerides**			0.057
No	0.90 (0.87, 0.93)	< 0.001	
Yes	0.86 (0.82, 0.91)	< 0.001	

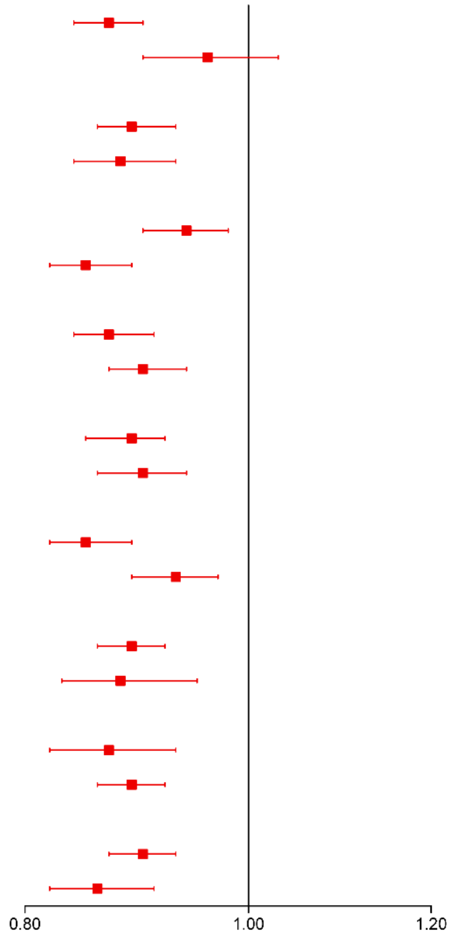

Supplemental Figure S2. Stratified analysis for the association between eGDR and liver cancer risk. Models were adjusted for age, sex, ethnicity, education, townsend deprivation index, physical activity, smoking status, alcohol consumption, sleep duration, BMI, central obesity, diabetes, low HDL, and high TG. Levels of significance: $p < 0.05$. eGDR, estimated glucose disposal rate; BMI, body mass index; HDL, high density lipoprotein; MASLD, metabolic dysfunction-associated steatotic liver disease; TG, triglyceride.

Subgroup	HR (95% CI)	P value	P for interaction
**Age, years**			0.790
<65	0.86 (0.77, 0.96)	0.010	
≥65	1.01 (0.85, 1.18)	0.948	
**Sex**			0.563
Male	0.91 (0.82, 1.02)	0.092	
Female	0.90 (0.74, 1.10)	0.318	
**Central obesity**			0.090
No	0.98 (0.85, 1.12)	0.758	
Yes	0.84 (0.73, 0.96)	0.012	
**Physical activity**			0.775
Enough activity	0.91 (0.80, 1.04)	0.171	
Not meeting enough activity	0.91 (0.79, 1.04)	0.165	
**Somking**			0.250
Never	0.83 (0.69, 1.00)	0.051	
Previous/current	0.94 (0.84, 1.05)	0.289	
**Alcohol consumption**			0.063
No heavy alcohol consumption	0.86 (0.75, 0.98)	0.022	
Heavy alcohol consumption	0.97 (0.84, 1.12)	0.697	
**Diabetes**			0.953
No	0.91 (0.81, 1.02)	0.121	
Yes	0.88 (0.73, 1.05)	0.153	
**Low HDL**			0.551
No	1.07 (0.84, 1.37)	0.580	
Yes	0.88 (0.80, 0.98)	0.019	
**High triglycerides**			0.263
No	0.90 (0.81, 1.01)	0.065	
Yes	0.92 (0.75, 1.11)	0.376	

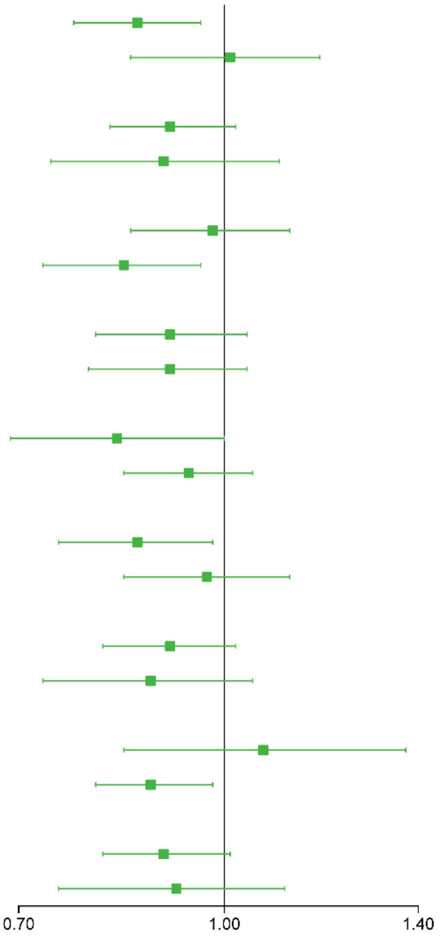

Supplemental Figure S3. Stratified analysis for the association between eGDR and liver-related mortality risk. Models were adjusted for age, sex, ethnicity, education, townsend deprivation index, physical activity, smoking status, alcohol consumption, sleep duration, BMI, central obesity, diabetes, low HDL, and high TG. Levels of significance: $p < 0.05$. eGDR, estimated glucose disposal rate; BMI, body mass index; HDL, high density lipoprotein; MASLD, metabolic dysfunction-associated steatotic liver disease; TG, triglyceride.

**Subgroup**	**HR (95% CI)**	**P value**	**P for interaction**
**Age, years**			0.498
<65	0.89 (0.78, 1.02)	0.093	
≥65	0.93 (0.76, 1.14)	0.499	
**Sex**			0.745
Male	0.93 (0.82, 1.06)	0.259	
Female	0.82 (0.64, 1.05)	0.115	
**Central obesity**			0.025
No	1.04 (0.87, 1.23)	0.694	
Yes	0.81 (0.69, 0.94)	0.007	
**Physical activity**			0.917
Enough activity	0.89 (0.74, 1.05)	0.168	
Not meeting enough activity	0.93 (0.79, 1.08)	0.320	
**Somking**			0.168
Never	0.66 (0.51, 0.87)	0.003	
Previous/current	0.98 (0.87, 1.12)	0.795	
**Alcohol consumption**			0.035
No heavy alcohol consumption	0.80 (0.69, 0.92)	0.002	
Heavy alcohol consumption	1.04 (0.89, 1.23)	0.606	
**Diabetes**			0.733
No	0.93 (0.82, 1.07)	0.308	
Yes	0.82 (0.65, 1.02)	0.076	
**Low HDL**			0.129
No	0.84 (0.56, 1.25)	0.385	
Yes	0.91 (0.81, 1.03)	0.131	
**High triglycerides**			0.488
No	0.90 (0.79, 1.03)	0.130	
Yes	0.90 (0.72, 1.12)	0.358	

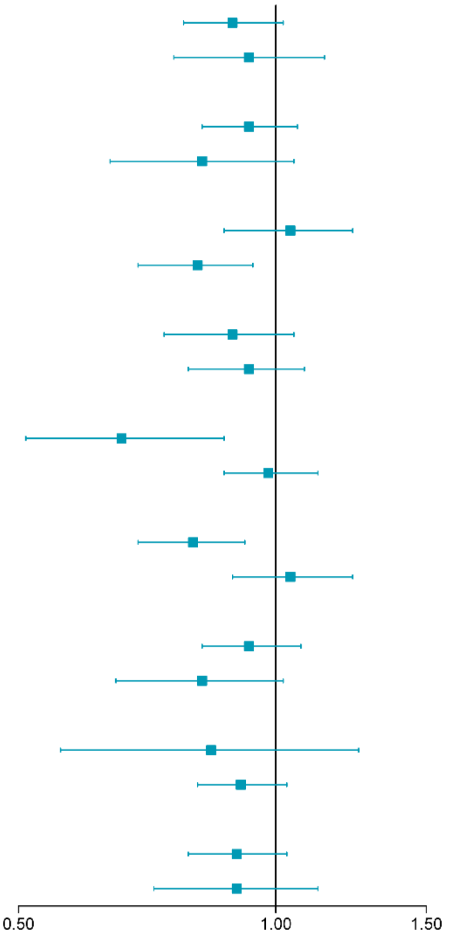

Supplement: Supplementary file 1 [file Data_Sheet_1.PDF]
